# Traditional Chinese medicine for symptoms of upper respiratory tract of COVID-19: A protocol for systematic review and meta-analysis

**DOI:** 10.1097/MD.0000000000021320

**Published:** 2020-07-24

**Authors:** Fangqi Liang, Lei Dong, Li Zhou, Yu Shi, Li Tian

**Affiliations:** a Department of Otolaryngology, Hospital of Chengdu University of Traditional Chinese Medicine; b Department of Rehabilitation, the people's hospital of Wenjiang; c College of Acupuncture and Moxibustion and Tuina, Chengdu University of Traditional Chinese Medicine, China.

**Keywords:** COVID-19, systematic review, traditional Chinese medicine

## Abstract

**Background:**

Assessing the effectiveness and safety of traditional Chinese medicine (TCM) for symptoms of upper respiratory tract of coronavirus disease 2019 is the main purpose of this systematic review protocol.

**Methods:**

The following electronic databases will be searched from inception to Sep 2020: the Cochrane Central Register of Controlled Trials (CENTRAL), PubMed, EMBASE, Web of Science, TCM, China National Knowledge Infrastructure, Chinese Biomedical Literature Database, Chinese Scientific Journal Database (VIP database), and Wan-Fang Database. Search dates: from inception dates to June 2020. Language: English. Publication period: from inception dates to June 2020. The primary outcome is the time and rate of appearance of main symptoms (including coughing, pharyngalgia, and nasal obstruction). The secondary outcome is the length of hospital stay. Two independent reviewers will conduct the study selection, data extraction and assessment. RevMan V.5.3 will be used for the assessment of risk of bias and data synthesis.

**Results:**

The results will provide a high-quality synthesis of current evidence for researchers in this subject area.

**Conclusion:**

The conclusion of our study will provide an evidence to judge whether TCM is effective and safe for the patients with symptoms of upper respiratory tract of coronavirus disease 2019.

**Ethics and dissemination:**

This protocol will not evaluate individual patient information or affect patient rights and therefore does not require ethical approval. Results from this review will be disseminated through peer-reviewed journals and conference reports.

**PROSPERO registration number:**

CRD42020187422.

## 1 Introduction

Coronavirus disease 2019 (COVID-19) is an emerging disease with a rapid increase in cases and deaths since its first identification in Wuhan, China, in December 2019.^[1]^ COVID-19 is caused by a new found coronavirus called SARA-CoV-2, previously known as 2019-nCoV. This kind of virus is in the same betacoronavirus clade as Middle East respiratory syndrome CoV (MERS-CoV) and the severe acute respiratory syndrome CoV (SARS-CoV) in relative genomic researches,^[2]^ which means 2019-nCoV may share the same infection mechanisms with the other 2, and it may be cured with the same interventions which are effective for MERS-CoV and SARS-CoV. COVID-19 had a great influence on people's health and lives, resulting 80,270 laboratory and clinical confirmed cases, and 2981 deaths in the mainland as of 24:00 on 3 March 2020. There are a plenty of methods to diagnose the COVID-19, of which nucleic acid test is the best choice.^[3]^ COVID-19 could cause various symptoms, of which respiratory distress is the most common one. Most of the patients with COVID-19 could not breath spontaneously, which is life-threatening.^[4]^ The respiratory distress, especially the upper respiratory track symptoms like sore throat and coughing, is caused by the replication of viruses.^[5]^

Currently, there are no specific antiviral drugs or vaccines for the COVID-19, which make symptomatic treatment the main strategies in clinical practice.^[6]^ Traditional Chinese medicine (TCM) belongs to the complementary and alternative medicine, which plays an important role in the preventing, treating, and curing of diseases and maintaining of health in China since 200AD.^[7]^ With its unique curative effect, TCM has been spreading over the world in recent years. What is more, TCM has shown a great help in the treatment and prevention of COVID-19 in 2020.^[8]^ The prescription, consist of herbs, could play an overall regulatory effect via multi-component and multi-target in network pharmacology analysis. And functional enrichment analysis showed that Chinese medicine could inhibit and alleviate excessive immune response and eliminate inflammation by regulating immune related pathway and cytokine action related pathway.^[9]^

This review aims to systematically evaluate the effectiveness and safety of TCM for symptoms of upper respiratory tract of COVID-19 by including multiple clinical trials published over the past 10 years.

## 2 Methods and analysis

### 2.1 Study registration

This systematic review protocol was registered with PROSPERO 2019 (registration number: CRD42020187422). And the protocol report is in the base of the Preferred Reporting Items for Systematic Reviews and Meta-Analyses Protocols (PRISMA-P) declaration guidelines.^[10]^ The review will be performed in line with the PRISMA declaration guidelines.^[11]^

### 2.2 Inclusion criteria for study selection

#### 2.2.1 Type of study

All randomized controlled trials (RCTs) about TCM for symptoms of upper respiratory tract of COVID-19 which were reported in English and Chinese will be included. Trials with two-arm or three-arm parallel design will be also included. Non-RCTs, quasi-RCTs, case series, reviews, animal studies, and any study with a sample size of less than 10 participants will be excluded.

#### 2.2.2 Type of participant

Patients with symptoms of upper respiratory tract of COVID-19, regardless of sex, age, race, or educational and economic status, will be included in the review.

#### 2.2.3 Type of interventions

Experimental interventions include TCM therapy. Control interventions would be western medicine therapy.

#### 2.2.4 Type of outcome measures

The primary outcome is the time and rate of appearance of main symptoms (including coughing, pharyngalgia, and nasal obstruction). The secondary outcome is the length of hospital stay.

### 2.3 Search methods for identification of studies

#### 2.3.1 Electronic data sources

The following electronic databases will be searched from inception to Sep 2020: the Cochrane Central Register of Controlled Trials (CENTRAL), PubMed, EMBASE, Web of Science, TCM, China National Knowledge Infrastructure, Chinese Biomedical Literature Database, Chinese Scientific Journal Database (VIP database), and Wan-Fang Database. The language will be limited to Chinese and English.

#### 2.3.2 Searching other resources

The reference lists of potentially missing eligible studies will be scanned ant the relevant conference proceedings will be scanned as well.

### 2.4 Search strategy

The search strategy for PubMed is shown in Table 1. The following search keywords will be used: TCM (eg, “Chinese Drugs, Plant” or “Chinese Herbal Drugs” or “Herbal Drugs, Chinese” or “Plant Extracts, Chinese” or “Chinese Plant Extracts” or “Extracts, Chinese Plant”); Respiratory Tract Infections (eg, “Infection, Respiratory Tract” or “Respiratory Tract Infection” or “Infections, Respiratory” or “Respiratory Infections” or “Infections, Respiratory Tract” or “Upper Respiratory Tract Infections” or “Infections, Upper Respiratory Tract” or “Infections, Upper Respiratory” or “Respiratory Infection, Upper” or “Upper Respiratory Infections”); COVID-19 (eg, “2019-nCoV” or “Wuhan coronavirus” or “SARS-CoV-2” or “2019 novel coronavirus” or “COVID-19 virus” or “coronavirus disease 2019 virus” or “COVID19 virus” or “Wuhan seafood market pneumonia virus”); randomized controlled trial (eg, “randomized controlled trial” or “controlled clinical trial” or “random allocation” or “randomized” or “randomly” or “double-blind method” or “single-blind method” or “clinical trial.” The equivalent search keywords will be used in the Chinese databases.

**Table 1:**
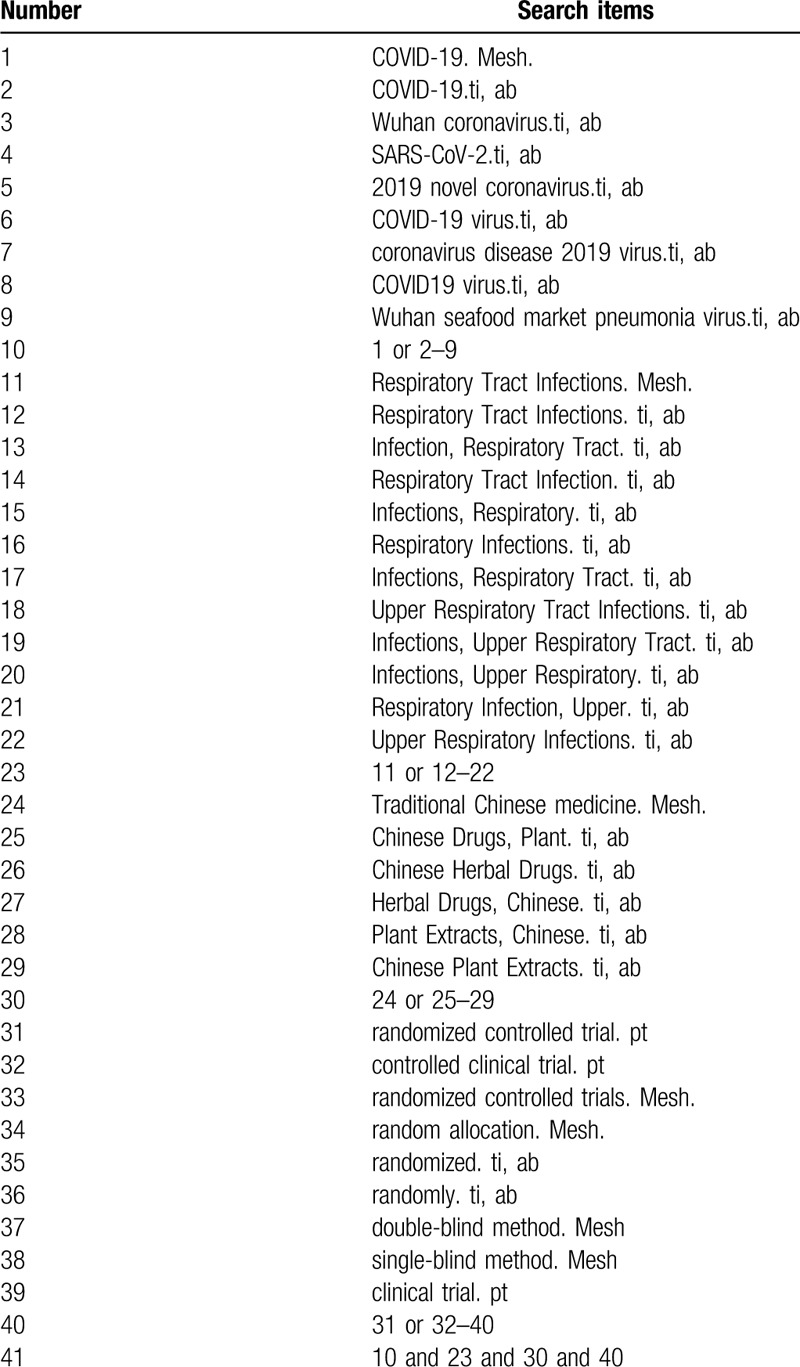
Search strategy for the PubMed database.

### 2.5 Data collection and analysis

#### 2.5.1 Selection of studies

**Figure 1:**
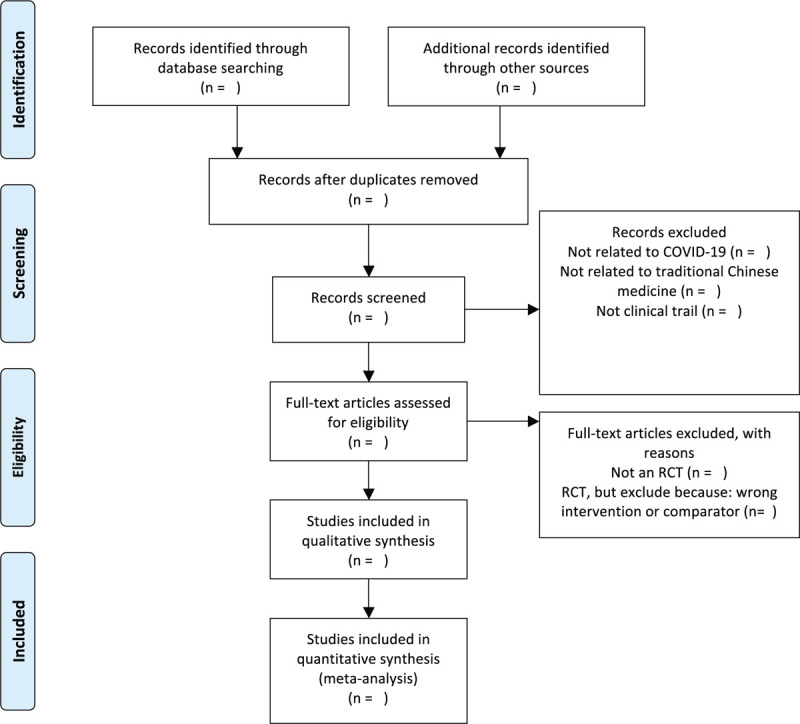
The PRISMA-P flow chart of the study selection process.

Two trained reviewers will review and screen the titles and abstracts of all searched studies independently. After eliminating duplicate records and ineligible studies, the full text of eligible studies will be reviewed to determine whether they meet the predefined inclusion criteria. Where the researchers are unable to reach a consensus, a third reviewer will make the final judgement. A PRISMA flow diagram will be used to show the study selection process in Figure 1.

### 2.6 Data extraction and management

The following data will be extracted from the selected studies by 2 independent reviewers using a standard data extraction sheet: year of publication, country, general information, participant characteristics, inclusion and exclusion criteria, sample size, randomization, blinding methods, methods, type of acupuncture interventions, control, outcome measures, results, adverse reactions, conflicts of interest, ethical approval, and other information. The authors will be contacted for further information if the reported data is insufficient and the third reviewer will be set to solve the disagreements.

### 2.7 Assessment of risk of bias and reporting of study quality

Two independent reviewers will access the quality of included literature and complete the Standards for Reporting Interventions in Clinical Trials of Acupuncture (STRICTA) checklist with the Cochrane collaboration risk-of-bias assessment method.^[12]^

### 2.8 Measures of treatment effect

For dichotomous data, the risk ratio with 95% confidence intervals (CIs) will be used for analysis. For continuous data, the mean difference with 95% CIs will be used. Standardized mean differences with 95% CIs will be used if different scales were used to measure a certain outcome variable.

### 2.9 Unit of analysis issues

The individual participant will the analytical unit.

### 2.10 Management of missing data

The cause of the missing data will be determined to solve the problem. And if this is not working, the authors will be contacted for the missing part. This will be documented and the available data will be extracted and analyzed if the missing data cannot be obtained.

### 2.11 Assessment of heterogeneity

The standard χ^2^ test will be used to detect statistical heterogeneity, with the I^2^ test to quantify inconsistency. We will use the fixed-effects model if the *P* value exceeds 0.1, and the I^2^ value is less than 50%, which indicates that studies are homogeneous. If the *P* value is less than 0.1, or the I^2^ value exceeds 50%, studies will be considered to have significant statistical heterogeneity, and subgroup analysis will be performed to explore the possible cause; if the heterogeneity remains significant, the random-effects model will be used.

### 2.12 Assessment of reporting bias

Funnel plots will be used to access the reporting biases if there are over 10 trials included in the meta-analysis [Higgins JP, Green S. ∗Cochrane handbook for systematic reviews of interventions∗: Wiley Online Library, 2008.].

### 2.13 Data synthesis

RevMan V.53 will be used for data synthesis. The level of statistical heterogeneity will determine how the data will be synthesized and analyzed. The random-effects model will be used if the I^2^ value is no less than 50%. The fixed-effects model will be used if the heterogeneity tests show little statistical heterogeneity. If there is meaningful heterogeneity that cannot be explained by any assessment, meta-analysis will not be performed. If necessary, each subgroup will be carefully considered for subgroup analysis.

### 2.14 Subgroup analysis

Subgroup analysis will be conducted if data are available. Factors such as different types of control interventions and different outcomes will be considered.

### 2.15 Sensitivity analysis

Sensitivity analysis will be conducted to test the robustness of the review conclusions if possible. The impacts of sample size, study design, methodological quality, and missing data will be evaluated.

### 2.16 Grading of evidence quality

The Grading of Recommendations Assessment approach will be applied to judge the quality of the evidence for all outcomes.^[13]^ Limitation of study design, inconsistency of results, indirectness, imprecision, and publication bias will be assessed. The assessments will be classified into 4 levels: high, moderate, low, or very low.

### 2.17 Ethics and dissemination

This protocol will not evaluate individual patient information or affect patient rights and therefore does not require ethical approval. Results from this review will be disseminated through peer-reviewed journals and conference reports.

## 3 Discussion

This systematic review will be the first to assess the effectiveness and safety of TCM for symptoms of upper respiratory tract of COVID-19, and its results will address a gap in the literature. The review contains four sections: identification, study inclusion, data extraction, and data synthesis. This review will aid doctors in the decision-making process for treating patients with symptoms of upper respiratory tract of COVID-19, and will provide information for patients and health policy makers.

## Author contributions

FQL, LD and LZ mainly contributed to this manuscript and joint first authors. LT obtained funding. FQL, LD and LZ drafted the protocol. LZ and YS make the search strategy and it will be conducted by them. LD and LZ will obtain copies of the studies and FQL and YS will screen the studies to be included. Data extraction from the studies will be done by LD and YS. FQL and LZ will put the data into RevMan. Analyses will be conducted by YS and LD will interpret the them. FQL, LD and LZ will draft the final review. LT will act as an arbiter in the study selection stage. All authors have read and approved the final manuscript.
